# Inhibition of TRPC4 channel activity in colonic myocytes by tricyclic antidepressants disrupts colonic motility causing constipation

**DOI:** 10.1111/jcmm.17348

**Published:** 2022-05-12

**Authors:** Byeongseok Jeong, Tae Sik Sung, Dongju Jeon, Kyu Joo Park, Jae Yeoul Jun, Insuk So, Chansik Hong

**Affiliations:** ^1^ Department of Physiology Chosun University School of Medicine Gwangju South Korea; ^2^ Department of Surgery Seoul National University College of Medicine Seoul National University Hospital Seoul South Korea; ^3^ Biomedical Research Institute Seoul National University Hospital Seoul South Korea; ^4^ 37990 Department of Physiology and Institute of Dermatological Science Seoul National University College of Medicine Seoul South Korea

**Keywords:** irritable bowel syndrome, m*I*_cat_, smooth muscle, tricyclic antidepressant, TRPC4

## Abstract

Tricyclic antidepressants (TCAs) have been used to treat depression and were recently approved for treating irritable bowel syndrome (IBS) patients with severe or refractory IBS symptoms. However, the molecular mechanism of TCA action in the gastrointestinal (GI) tract remains poorly understood. Transient receptor potential channel canonical type 4 (TRPC4), which is a Ca^2+^‐permeable nonselective cation channel, is a critical regulator of GI excitability. Herein, we investigated whether TCA modulates TRPC4 channel activity and which mechanism in colonic myocytes consequently causes constipation. To prove the clinical benefit in patients with diarrhoea caused by TCA treatment, we performed mechanical tension recording of repetitive motor pattern (RMP) in segment, electric field stimulation (EFS)‐induced and spontaneous contractions in isolated muscle strips. From these recordings, we observed that all TCA compounds significantly inhibited contractions of colonic motility in human. To determine the contribution of TRPC4 to colonic motility, we measured the electrical activity of heterologous or endogenous TRPC4 by TCAs using the patch clamp technique in HEK293 cells and murine colonic myocytes. In TRPC4‐overexpressed HEK cells, we observed TCA‐evoked direct inhibition of TRPC4. Compared with TRPC4‐knockout mice, we identified that muscarinic cationic current (m*I*_cat_) was suppressed through TRPC4 inhibition by TCA in isolated murine colonic myocytes. Collectively, we suggest that TCA action is responsible for the inhibition of TRPC4 channels in colonic myocytes, ultimately causing constipation. These findings provide clinical insights into abnormal intestinal motility and medical interventions aimed at IBS therapy.

## 1 INTRODUCTION

The function of the gastrointestinal (GI) tract is to ingest, digest, and absorb nutrients and eliminate waste. GI motor patterns in mammalian are largely composed of colonic motor complexes (CMCs), a mass movement, and spontaneous phasic contractions called ripples.^1^, ^2^ In order to generate these repetitive propagating sequences in GI motility, motor complexes in smooth muscle layer require harmonized coordination between enteric neurons, interstitial cells of Cajal (ICCs) and smooth muscle cells (SMCs).^3^, ^4^ The enteric nervous system (ENS) includes intrinsic neural plexuses and autonomic extrinsic neural pathways, which are of fundamental importance for generating major motor patterns and regulating the amplitudes of contractions.^5^, ^6^ The polarized enteric circuits conducted to intramuscular ICC (ICC‐IM) and SMCs generate CMCs.^3^, ^7^ And spontaneous electrical activity generated by myenteric ICC (ICC‐MY) is conducted to SMCs which consequently leads to slow wave and spontaneous muscle contractions.^3^, ^8^ The mechanical role of spontaneous contractions is controversial. Ehrlein et al.^9^ suggested that they were mainly involved in mixing rather than propulsion. On the contrary, it has been argued that spontaneous contractions are not effective contractions for mixing and propulsion by themselves, but they cause larger contractions when they occur concurrently with other contractions.^2^, ^10^ Repetitive motor patterns (RMPs), defined only in human GI motility, are complex propulsive contractions triggered by enteric nerve stimulation or spontaneous.^2^ The smooth muscle response to external signals ultimately depends on the excitability of the syncytium consisting of ICCs and SMCs.^11^, ^12^ Precise and balanced regulation of parasympathetic signalling is essential for GI motility; however, some medications, particularly antidepressants, can produce anticholinergic effects with constipation.^13^, ^14^

Antidepressants, which are the standard drug therapy used in the treatment of depression, typically act to restore the balance of neurotransmitter levels. Commonly prescribed antidepressants, such as tricyclic antidepressants (TCAs), selective serotonin reuptake inhibitors (SSRIs) and serotonin‐norepinephrine reuptake inhibitors, have different mechanisms of action and consequent pleiotropic effects. TCAs, which consist of a 3‐ring in their core chemical structure, include amitriptyline (AMI; Elavil), imipramine (IMI; Tofrnail), doxepin (Adapin) and desipramine (DES; Norpramin). Previously, most evidences have indicated that TCA antagonizes histamine H1, adrenoreceptor and muscarinic acetylcholine receptor, directly producing a cluster of symptoms called anticholinergic side effects, which include sedation, drowsiness, postural hypotension, blurred vision, dry mouth and constipation.^15^ Although SSRIs are a first‐line antidepressant because they have potentially fewer side effects,^16^, ^17^ the clinical value of TCAs still exists not only in neuropathic pain^18^, ^19^ and Parkinson's disease (PD)^20^ but also in irritable bowel syndrome (IBS).^21^, ^22^

One of the functional GI disorders, IBS, is accompanied by abdominal pain and abnormal stool form. IBS is classified into predominant stool patterns, such as IBS with diarrhoea (IBS‐D), IBS with constipation (IBS‐C) and mixed IBS (IBS‐M).^23^ Treatment is aimed at relieving pain and improving bowel problems, but addressing each individual's specific symptoms depending on the severity of symptoms is more important. The first‐line medical therapies for IBS are those that use laxative, antidiarrhoeal, and antispasmodic agents, but most randomized controlled trials could hamper personalized treatment based on the predominant symptoms.^24^ When the report that antidepressants had potential as a treatment for IBS was first presented three decades ago, SSRIs and TCAs were prescribed for the purpose of stabilizing the central nervous system (CNS). Since then, clinical evaluations of TCA have revealed not only central neuromodulation but also reduced GI motility. In addition, Siproudhis and colleagues demonstrated that AMI could be used to reduce the pressure of defecation by relaxing the anal sphincter muscle.^25^ Based on these findings, TCA prescription for patients with IBS is considered suitable, especially for patients with predominant pain and diarrhoea (IBS‐D).

Although, to date, there are several preclinical experimental studies that have investigated the efficacy of TCAs, the identification of on‐ and off‐targets is needed to overcome some of the potential limitations of TCAs. Duncan et al.^26^ found that doxepin, similar to other TCAs, caused long QT interval prolongation by inhibiting hERK channels of ventricular myocytes from rabbits. Dennis et al.^27^ identified that, in addition to blockade of the hERG current, trafficking inhibition and degradation of hERG are responsible for the cardiotoxicity of TCAs. Hamaguchi et al.^28^, ^29^ reported that the inhibitory effect of IMI on the TRPM‐like channels contributes to vascular smooth muscle homeostasis by reducing Mg^2+^ influx in the porcine carotid artery. On the basis of a wide area of pain and symptoms caused by TCAs, we focus on the potential role of transient receptor potential canonical (TRPC) proteins, ubiquitously expressed in the nervous, digestive and reproductive systems.^30^, ^31^ In intestinal smooth muscle cells isolated from knockout mice, TRPC4 and TRPC6 channels, gated by muscarinic receptors, are already well known to be responsible for muscarinic cationic current (m*I*_cat_).^32^ Based on pharmacological intervention that inhibits large intestinal motility to alleviate abdominal symptoms accompanied by diarrhoea,^33^ we evaluated whether the TRPC4 channel has potential as a clinical candidate.

In this study, we report on the inhibitory effect of the TRPC4 current by TCAs underlying the causality of colonic motility for TCA‐induced constipation using human and murine colons. Within the estimated serum concentration range of TCAs,^34^ the TRPC4 channel can have functional potential as an alternative molecular target for TCAs. These findings provide clinical insights into abnormal intestinal motility and medical interventions aimed at IBS therapy.

## 2 METHOD

### 2.1 Cell culture, transient transfection and chemicals

Human embryonic kidney (HEK293) cells were obtained from the American Type Culture Collection (ATCC). The cells were maintained according to the supplier's recommendations. For transient transfection, we used the transfection reagent Lipofectamine 3000 (Invitrogen) for molecular biology tools or FuGENE 6 (Promega) for electrophysiological experiments according to the manufacturer's protocol. All experiments were performed 20–30 h after transfection. All chemicals were purchased from Sigma Aldrich, while Carbachol (CCh) was purchased from Tocris.

### 2.2 Isolation of murine colonic myocytes

The animal experiments were approved by the ethics committee of Chosun University, according to the National Institutes of Health Guide for the Care and Use of Laboratory Animals (CIACUC 2020‐S0038). Colonic myocytes were isolated from 30‐ to 60‐day‐old C57BL/6N mice of either sex. Mice were anaesthetized with isoflurane and sacrificed by cervical dislocation, and the sigmoid colon was quickly isolated. The colon was opened along the myenteric border, and the mucosa and submucosa layer were removed in Ca^2+^‐free Hanks solution containing (in mM) 135 NaCI, 5 KCI, 5 glucose and 5 HEPES with the pH adjusted to 7.4 using NaOH. Strips of colonic muscle were transferred to the same solution with 0.1% collagenase (Worthington Biochemical Co.), 0.2% bovine serum albumin (Sigma), 0.1% trypsin inhibitor (Sigma) and 0.01% papain (Sigma). Incubation in the enzyme solution was carried out at 37°C for 10–15 min, and then the tissues were washed with Ca^2+^‐free Hanks solution. Single cells were obtained by gentle agitation with a wide‐bored glass pipette. Isolated cells were kept at 4℃ until use. Before electrophysiological experiments, a drop of the suspension was pipetted into a small chamber (0.3 ml) on the stage of an inverted microscope. All experiments were carried out within 3 h of cell dispersion and performed at room temperature.

### 2.3 Human tissue acquisition and tissue preparations

Human colon tissues were obtained from patients who underwent selective radical surgery for nonobstructive bowel cancer. This study was approved by the Institutional Review Board (IRB) of the Seoul National University Hospital (IRB approval number: H‐0603‐071‐170). The study protocol was performed in accordance with the guidelines and regulations of the SNUH IRB. Written informed consent was received from all patients before the operations. After anterior resection, sigmoid colon specimens were obtained from patients without previous chemoradiotherapy. Specimens were immediately immersed in preoxygenated Krebs‐Ringer bicarbonate (KRB) solution containing (in mM) 120.4 NaCl, 15.5 NaHCO_3_, 5.9 KCl, 11.5 glucose, 1.2 NaH_2_PO_4_, 2.5 CaCl_2_ and 1.2 MgCl_2_. This solution was adjusted to pH 7.3–7.4 at 37°C and equilibrated with 97% O_2_ and 3% CO_2_.^35^, ^36^

### 2.4 Mechanical tension recordings

Whole colonic segments with intact mucosal layers were dissected parallel to the longitudinal muscle using a pair of scissors.^35^, ^36^ To mimic natural colonic segment, a flat dissected colon was reformed to tubular shape. Circular muscle tension of each segment (5 cm in length and 2 cm in width) was recorded at three sites (proximal, middle and distal sites, 2 cm apart) via perpendicular traction using sutures placed at each site. Sutured muscle was connected to an isometric force transducer (Biopac Systems) using threaded stainless steel micro serrefines (Fine Science Tools). Colonic segments were equilibrated for at least 2 h before experiments under a resting force of 1 g. Prewarmed (36.5 ± 0.5°C) and preoxygenated KRB solution was perfused continuously into the tissue chamber. The AUC for 10 min was analysed for RMPs before and after the application of drugs. The mechanical responses were recorded and digitized using Acknowledge software (Biopac Systems). Data were analysed offline using Clampfit (version 10.7. Molecular devices).

For recording spontaneous contractions, colonic muscle strips (6 mm in length and 2 mm in width) without the mucosal layers were dissected parallel to the circular muscle layer using a knife consisting of double parallel scalpel blades set 1.5 mm apart.^36^ The remnant muscle strips were connected to an isometric force transducer (Biopac Systems) and suspended in a 10‐ml organ bath containing prewarmed (36.5 ± 0.5°C) and preoxygenated KRB solution. The muscle strips were stabilized for 60 min without a force followed by equilibration for 60 min under a resting force of 1 g. Electrical field stimulation (EFS; 1–16 Hz, 10 s, 100 V) was applied to evoke contractions in the presence of L‐NNA (100 μM) and MRS 2500 (1 μM) to eliminate inhibitory responses. The amplitude was analysed for EFS‐induced contraction, and the area under the curve (AUC) for 5 min was analysed for spontaneous contractions before and after the application of drugs. The mechanical responses were recorded and digitized using Acknowledge software (Biopac Systems). Data were analysed offline using Clampfit (version 10.7. Molecular devices). Tetrodotoxin (TTX, 1 μM) was administered for 10 min before the application of TCAs to eliminate neural involvement in TCA‐induced responses in some experiments. The methods described above are similar to those in previous studies.^36^, ^37^

### 2.5 Electrophysiology for recording of TRPC4 current

Whole‐cell currents were recorded using an Axopatch 200B amplifier (Axon Instruments), Digidata 1550B Interface (Axon Instruments), and analysed with OriginPro 8 (OriginLab Co.). For whole‐cell experiments, glass capillaries (Harvard Apparatus) made with a resistance of 3–4 MΩ using a Narishige PC‐10 puller were filled with standard intracellular solutions containing (in mM): 140 CsCl, 10 HEPES, 0.2 Tris‐GTP, 0.5 EGTA and 3 Mg‐ATP with the pH adjusted to 7.3 using CsOH. We used an external bath solution (normal Tyrode solution) containing (in mM): 135 NaCl, 5 KCl, 2 CaCl_2_, 1 MgCl_2_, 10 glucose and 10 HEPES with the pH adjusted to 7.4 using NaOH. Transfected cells were trypsinized and transferred into a recording chamber equipped to be treated with a number of solutions. Cs^+^‐rich solution was made by replacing monovalent cation (NaCl and KCl) in normal tyrode with equimolar CsCl. Voltage ramp pulses were applied from +100 to −100 mV for 500 ms at a holding potential of −60 mV. The current (I)‐voltage (V) curve is shown by roman numerals on the current trace. For all bar graphs, inward current amplitudes at −60 mV are summarized.

### 2.6 Surface biotinylation

The cells were lysed to extract the proteins using lysis buffer (1% Triton X‐100, 150 mM NaCl, 50 mM HEPES, 2 mM MgCl_2_, 2 mM EDTA, pH 7.4 and protease inhibitor cocktail (Roche)). After incubation at 4°C for 30 min, each sample was centrifuged at 13,000 *g* at 4°C for 15 min. Supernatants were collected, and protein concentration was measured at 750 nm using DC Protein Assay (BioRad). A defined quantity of total protein was electrophoresed on an 8% SDS‐polyacrylamide gel, and then subsequently transferred onto a nitrocellulose membrane. Each membrane was blocked using 5% BSA prepared in TBST. Each membrane was incubated at 4°C overnight with primary antibody diluted in 5% BSA prepared in TBST buffer. The proteins were probed with GFP (Life technology). β‐Tubulin (Sigma) antibodies were used as housekeeping proteins. Each membrane was incubated with secondary antibody for 1 h 30 min.

For surface biotinylation, washed twice with prechilled PBS cells were incubated in 0.5 mg/ml sulfo‐NHS‐SS‐biotin (Thermo Scientific) in PBS for 30 min on ice. Afterwards, the free biotin was quenched by the addition of 100 mM glycine in PBS. The cells were then processed as described above to make cell extract. 40 μl of 50% aqueous slurry of immobilized avidin beads (Thermo Scientific) was added to 400 μl of cell lysates (0.5–1 mg protein). After incubation with gentle shaking for 1 h at room temperature, beads were washed three times with 0.5% Triton‐X‐100 in PBS, and proteins were extracted in 5×sample buffer. Collected proteins were then analysed by Western blot. Na^+^/K^+^‐ATPase (NKA) was used as control of membrane protein detected by alpha 1 Sodium Potassium ATPase antibody (Abcam).

### 2.7 Statistics

Origin 8 (OriginLab Co.) or Prism 5.0 (GraphPad) software was used for all analyses. All results are given as the mean ± SEM. The results were compared using Student's *t*‐test for two groups or anova followed by post hoc test for three groups or more. *p* values < 0.05 were considered statistically significant. The number of electrical recordings and Western blots is given by n in the bar graph or figure legends. ^#^ *p* < 0.1 *p < 0.05, **p < 0.01 and ***p < 0.001 versus control.

## 3 RESULTS

### 3.1 TCA strongly inhibits the mechanical activity of human colonic smooth muscle

The prescription of TCAs with high anticholinergic activity exhibits a number of anticholinergic signs and symptoms, such as dry mouth, blurred vision, urinary retention, constipation and hallucinations.^38^, ^39^ In addition, in TCA overdose, there are likely symptoms that raise suspicion for cardiovascular toxicity, such as arrhythmias and refractory hypotension.^40^ Nevertheless, it has been shown to be extraordinarily beneficial to improve global IBS symptoms.^21^, ^41^ However, in the action of TCA for treating IBS by inhibiting GI motility, molecular candidates and the exact mechanism have not been clearly established.

**FIGURE 1:**
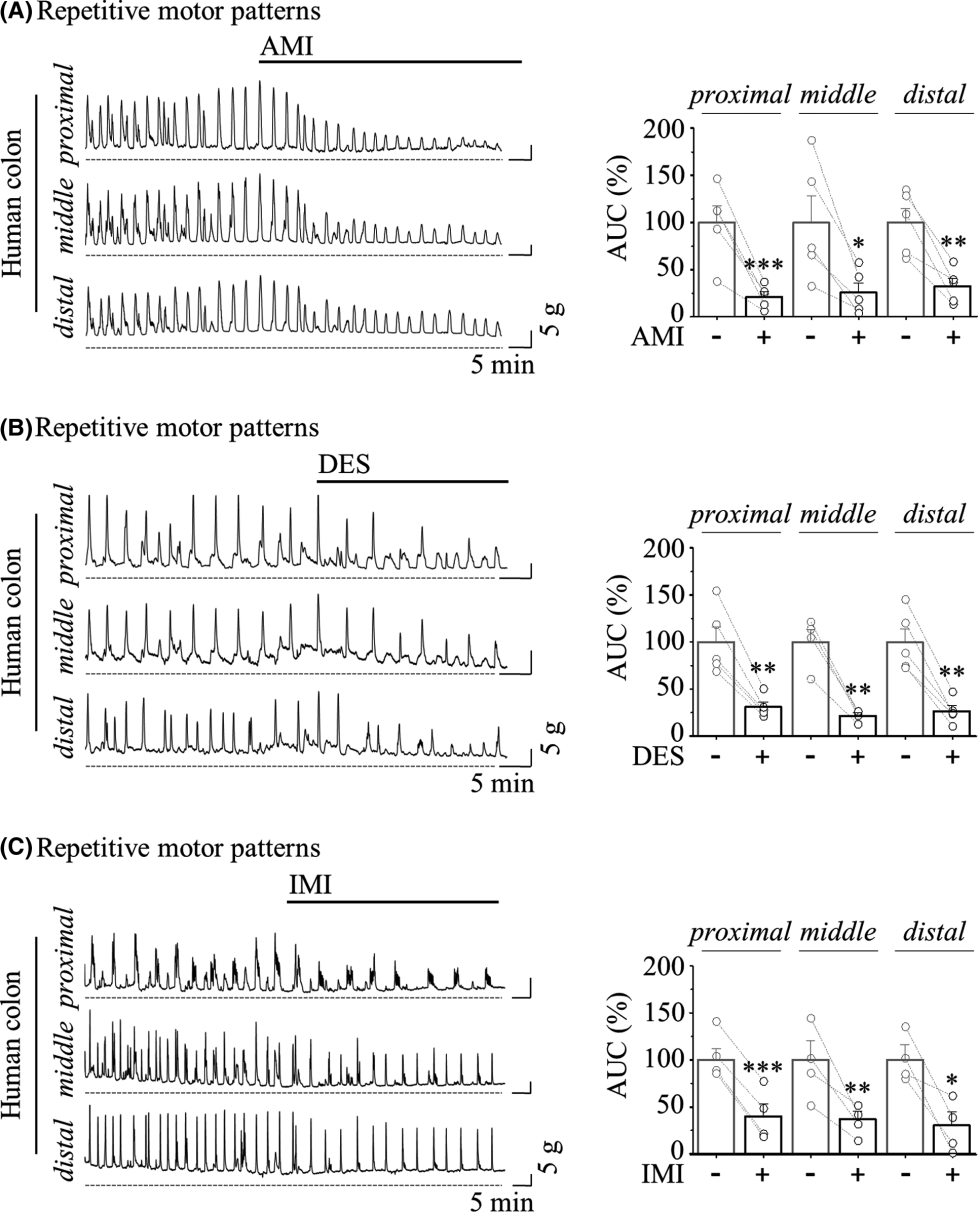
TCA‐induced inhibitory effect on MMC in the human sigmoid colon. In all panels, representative mechanical traces (*left*) showed that three types of TCAs induced MMCs in the human sigmoid colon at the proximal, middle and distal sites. Compounds were applied at times indicated by bars, and the baseline tension was indicated by dotted lines. The summarized bar graph (*right*) before (grey) and after (black) TCA treatment. (A) 10 μM AMI (*n* = 5). (B) 10 μM DES (*n* = 5). (C) 10 μM IMI (*n* = 4)

To experimentally prove the clinical benefit in patients with diarrhoea by TCA treatment, we isolated normal colonic specimens from patients with colorectal cancer. As in our previous studies using mechanical tension recording,^35^ we first observed whether TCA inhibits the motility of isolated colon segments. By the application of 10 μM TCAs in extracellular Krebs‐Ringer solution, all three sites (proximal‐to‐distal) of the sigmoid colon segments showed inhibition of repetitive motor activities. As shown in Figure 1A, AMI robustly reduced the amplitude of RMPs at the proximal (21.20 ± 5.38%), middle (25.81 ± 10.22%) and distal (32.62 ± 8.14%) sites by at least 70% compared with those before exposure to AMI. As mentioned, TCAs possessing a common polycyclic chemical structure [Figure S1A] are known to exhibit similar biological effects.^42^ DES and IMI also significantly reduced repetitive motor activities (DES; 30.98 ± 5.09% at proximal, 21.45 ± 3.10% at middle, 26.45 ± 5.89% at distal in Figure 1B, and IMI; 39.82 ± 13.03% at proximal, 36.79 ± 8.30% in middle, 30.07 ± 14.24% at distal in Figure 1C).

**FIGURE 2:**
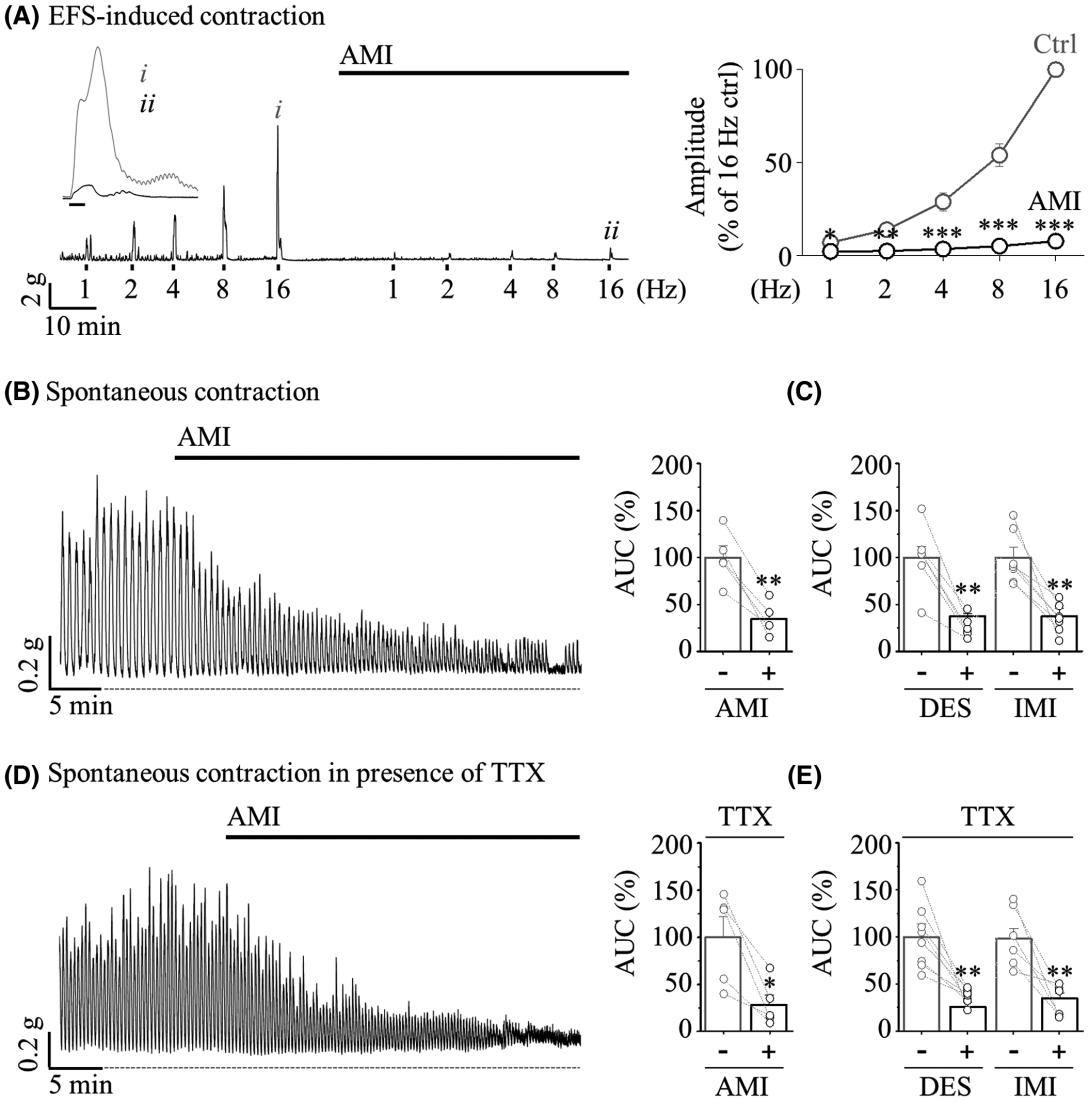
TCA‐induced inhibitory effect on EFS‐induced contraction and spontaneous contraction in human colonic muscle strips. (A) Representative traces (*left*) of EFS‐induced contraction suppressed by 10 μM AMI. Inset traces showing contractile responses to EFS at 16 Hz before (*i*) and after (*ii*) application of AMI. Summarized amplitude data (*right*) on the inhibition rate at 16 Hz (*n* = 10). (B–E) The representative traces (*left*) and summarized bar graph (*right*) of the human sigmoid colonic circular muscle strips before (grey) and after (black) TCA treatment. (B and D) 10 μM AMI (*n* = 5 of B, *n* = 5 of D). (C and E) 10 μM DES (*n* = 5 of C, *n* = 8 of E) or 10 μM IMI (*n* = 7 of C, *n* = 6 of E). (D and E) Pretreatment with 1 μM TTX for 10 min

Each ENS, smooth muscle (myocyte), and ICC cell type or all of these cell types might be responsible for the inhibition of RMP generation induced by TCA. To distinguish which cells TCA affects, we isolated the colon muscle strip in sigmoid colonic segments. And, excitatory muscular motor neurons were activated through EFS (1–16 Hz, 100 V) applied to the muscle strip in the presence of L‐NNA and MRS 2500. As shown in Figure 2A, EFS‐induced contractions were completely suppressed by AMI, and these contractile responses were gradually suppressed by DES or IMI at the highest frequencies (Figure S2A,B).

Although a colonic myocyte is the fundamental contractile unit of the colon and eventually myocytes should undergo excitation‐contraction coupling, whether neurogenic or myogenic, the functional importance of myocytes on GI motility is often underestimated. To determine whether TCA directly inhibits the electromechanical properties of the myocyte itself, we focused on spontaneous contraction of the circular muscle strip. At a concentration of 10 μM, AMI caused a gradual decrease in the ACU (34.64 ± 7.59%) of spontaneous contraction with no significant change in frequency compared with the basal level (Figure 2B). In pretreatment with 1 μM TTX to block neural stimulation input, spontaneous contractions before and after AMI treatment showed a similar pattern (28.30 ± 10.63%, Figure 2D). As shown in Figure S2C–F, DES and IMI also suppressed the AUC of spontaneous contractions (37.63 ± 2.83% of DES, 37.49 ± 8.40% of IMI without TTX pretreatment in Figure 2C, 26.11 ± 4.55% of DES, 35.23 ± 5.75% of IMI with TTX pretreatment in Figure 2E).

The results of Figures 1 and 2 indicated that RMPs, EFS‐induced contraction and spontaneous contraction were strongly inhibited by TCAs, suggesting that the clinical effects of TCA‐induced constipation and IBS‐D treatment with TCA are supported through our experimental findings.

### 3.2 TRPC4 channels closely contribute to the regulation of human colonic muscle contractions

**FIGURE 3:**
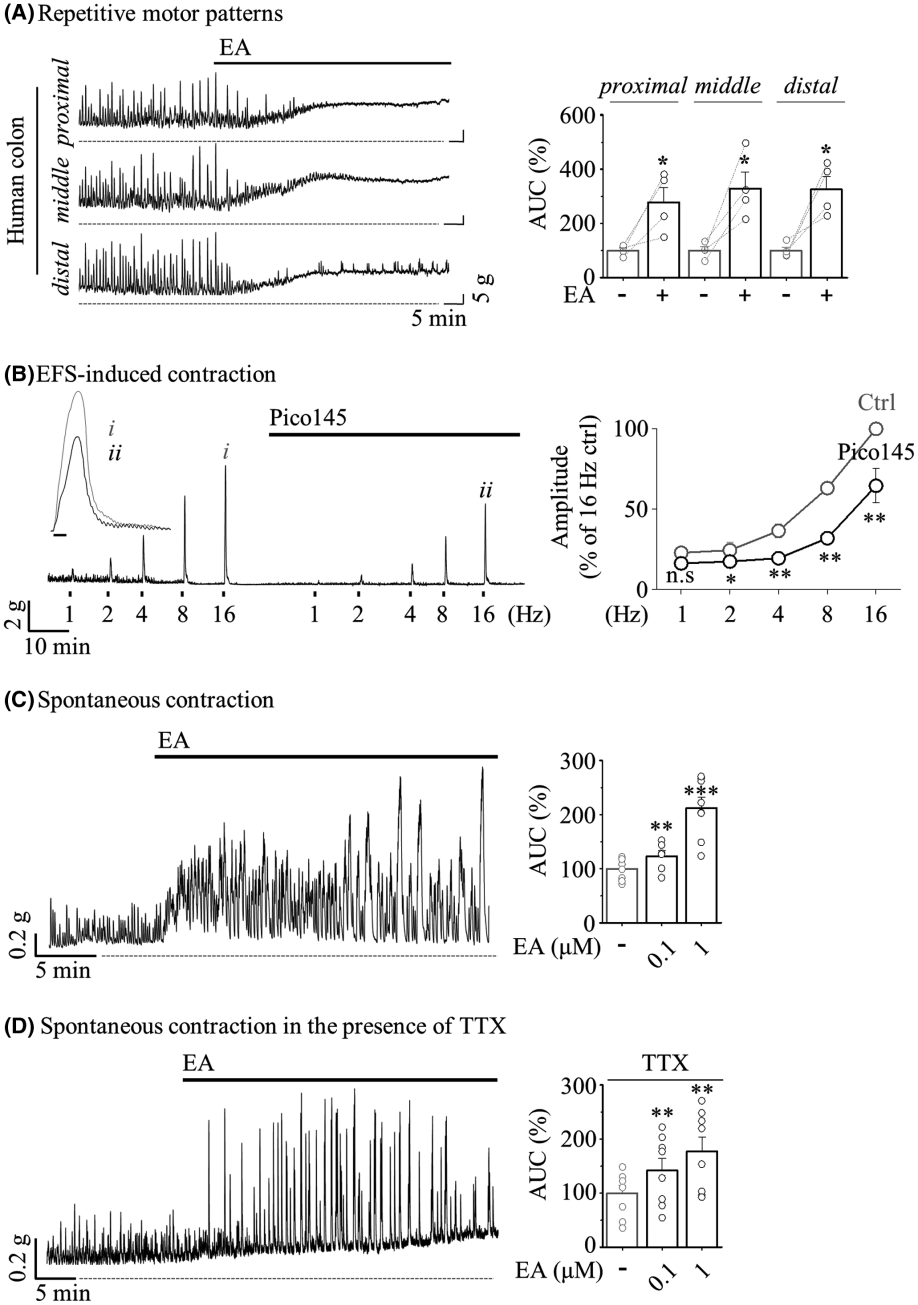
Effect of TRPC4 activation on human colonic muscle contraction. (A) Representative mechanical traces (*left*) of RMPs evoked by 1 μM EA. Summarized bar graph (*right*) before (grey) and after (black) EA application (*n* = 4). (B) Representative traces (*left*) of EFS‐induced contraction suppressed by 100 nM Pico145. Inset traces showing EFS‐induced amplitude before (*i*) and after (*ii*) application of Pico145. Summarized data (*right*) show the inhibition rate of amplitude (*n* = 4). (C and D) The representative traces (*left*) and summarized bar graph (*right*) before (grey) and after (black) EA application (*n* = 8 of vehicle, *n* = 6 of 0.1 μM and *n* = 8 of 1 μM in C). (D) Pretreatment with 1 μM TTX (*n *= 8)

We next attempted to identify the molecular candidate of TCA that inhibits colonic motility. Numerous studies have confirmed that TRPC4 channels in intestinal SMCs are gated by muscarinic receptors,^43^ and approximately 80% of m*I*_cat_ are mediated by TRPC4 activity.^32^ To determine the functional role of TRPC4 in GI motility using its pharmacological agonist or antagonist, we investigated RMP and EFS‐induced contractile activity as observed above. As shown in Figure 3A, 100 nM Englerin A (EA), a potent and selective activator of TRPC4, significantly increased repetitive motor activities. These sustained (tonic) contractions could result from smooth muscle,^44^ suggesting that TRPC4 has considerable potential for the depolarization of colonic myocytes. Tsvilovskyy et al.^32^ previously suggested that TRPC4 is indirectly activated by acetylcholine involved in neurogenic contraction. To rule out a contribution of TRPC4 to the neurogenic contraction, EFS‐induced contraction was compared in the absence or presence of Pico145 (a remarkable inhibitor of TRPC4). Pico145 (100 nM) was slightly suppressed by 64.64 ± 10.71% at the highest (16 Hz) frequencies (Figure 3B). Additionally, in the circular smooth muscle strip, Pico145 caused a substantial decrease in the AUC of spontaneous contraction rather than an amplitude of nearly half (Figure S3A). Conversely, EA dramatically increased the amplitude only of spontaneous contractions with tone (Figure 3C). EA‐enhanced spontaneous contractions were not altered by TTX (Figure 3D).

These results indicated that blockade of the TRPC4 channel induces atrophy not only in ENS‐mediated contractions but also in smooth muscle activation. Functional role of TRPC4 in neurogenic contraction should not be overlooked, but given that the dominant role of TRPC4 in the reports thus far is considered primarily to activate depolarization of intestinal myocytes, it is, therefore, considered to predominantly act to activate SMCs. These findings and suggestions indicated that TRPC4 is an essential determinant of colonic myocyte contraction causing intestinal motility. Thus, TRPC4 seems to be a reasonable candidate as a molecular target of TCA‐induced constipation and IBS treatment with TCA.

### 3.3 TCA evokes direct extracellular inhibition of TRPC4 channel activity

**FIGURE 4:**
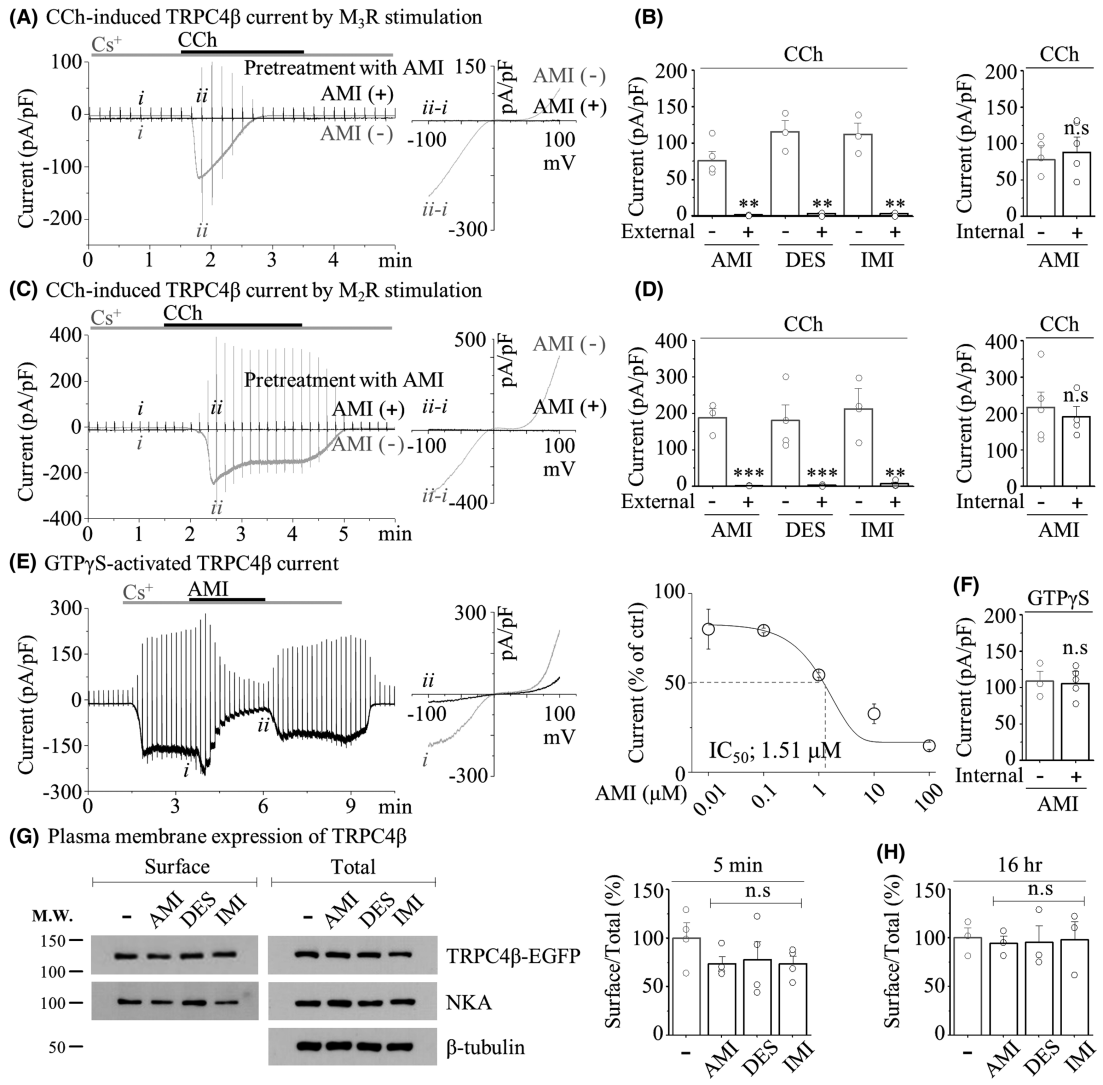
Inhibition of TRPC4 activity induced by extracellular TCA treatment. (A and C) Representative current trace (*left*) and current (I)–voltage (V) curve (*right*) showed TRPC4β current induced by M_3_R (A) or M_2_R (C) stimulation. Pretreatment with 10 μM AMI for 3 min before 100 μM CCh application. (B and D) Summarized bar graph of current density (pA/pF) by extracellular (*left*) or intracellular (*right*) treatment of 10 μM TCAs. (B) External solution (*left*) (*n* = 4 of AMI, *n* = 3 of DES and *n* = 3 of IMI) and internal solution (*right*) (*n* = 4 of vehicle and *n* = 4 of AMI). (D) External solution (*left*) (*n* = 3 of AMI, *n* = 4 of DES and *n* = 4 of IMI) and internal solution (*right*) (*n* = 5 of vehicle and *n* = 5 of AMI). (E) Representative current trace (*left*) and IV curve (*middle*) of 200 μM GTPγS‐activated current suppressed by 10 μM AMI. The dose‐dependent curve (*right*) of the inhibition rate depending on the AMI concentration. (F) Summarized bar graph of current density (pA/pF) by intracellular treatment of 10 μM AMI (*n* = 4 of vehicle, *n* = 5 of AMI). (G and H) Representative western blot (*left*) and quantified data of the ratio (*right*, H) of the PM expression level of TRPC4β‐GFP (*n* = 4 of 5 min and *n* = 3 of 16 h)

To investigate the electrical properties of the TRPC4 channel induced by TCA, we conducted patch clamp recordings in TRPC4‐overexpressing HEK293 cells. As mentioned above about the relevance of TRPC4 to altered electromechanical activity in colonic contraction induced by TCA, we expected that TCA inhibits TRPC4 channel activity. Since the stimulation of the muscarinic acetylcholine receptor elicits m*I*_cat_ for initiating cholinergic contraction, we measured the TRPC4 current by coexpression with muscarinic acetylcholine receptor type 2 (M_2_R) and type 3 (M_3_R), which are mainly expressed in smooth muscle.^45^, ^46^ As the Gα_q_‐PLC pathway is a primary activation of the TRPC4 channel, CCh stimulates M_3_R,^47^, ^48^ apparently showing a typical doubly rectifying TRPC4 current by M_3_R stimulation (Figure 4A). Pretreatment with 10 μM AMI completely inhibited the CCh‐activated TRPC4 current (75.56 ± 12.92 to 1.05 ± 0.25 pA/pF). DES (115.23 ± 15.12 to 3.05 ± 1.00 pA/pF) and IMI (111.77 ± 15.30 to 2.74 ± 1.35 pA/pF) also showed a remarkable inhibition of inward current (Figure 4B). Our group previously reported that the Gα_i2_ protein can directly activate the TRPC4 channel.^49^ When coexpressed with M_2_R, all TCA compounds completely inhibited the CCh‐induced TRPC4 current (AMI; 187.50 ± 24.87 to 1.05 ± 0.25 pA/pF, DES; 180.02 ± 42.91 to 2.74 ± 1.35 pA/pF, and IMI; 212.30 ± 55.77 to 6.41 ± 3.72 pA/pF), similar to M_3_R (Figure 4C,D).

Since TCA produces anticholinergic effects, such as constipation, especially in the colon, we asked whether TCA directly inhibits TRPC4 channel activity in smooth muscle. As addressed in our previous experiments, TRPC4 activation with the Cs^+^ current could be clearly observed with a relatively high Cs^+^ permeability of TRPC channels^50^ when GTPγS in an internal solution is infused and Cs^+^‐rich external solution is perfused. Similar to TRPC4 inactivation by TCAs, even in CCh‐evoked activation, AMI significantly inhibited Cs^+^ current activation by 200 μM GTPγS (Figure 4C). To assess the potency of TCAs against direct inhibition of TRPC4, we calculated the half maximal inhibitory concentration (IC_50_) values by applying various concentrations of TCAs. In GTPγS‐evoked TRPC4 activity, the IC_50_ of AMI was approximately 1.51 μM (Figure 4D), and those of DES and IMI were 5.37 μM and 6.12 μM, respectively (Figure S4A,B). In contrast to the inhibition of the TRPC4 current by extracellular bath perfusion of TCAs (Figure 4A,C,E), intracellular infusions of AMI did not inhibit the current at all compared with vehicle controls (Figure 4B,D,F).

A previous report by Dennis et al. suggested that TCA compounds simultaneously block the hERG current and its surface expression by promoting ubiquitination and degradation^27^; thus, we needed to validate this possibility on the TRPC4 channel using a surface biotinylation method. Preincubation with TCAs for a short exposure (5 min) or even for over 16 h overnight did not show any change in the expression level of TRPC4 protein on the plasma membrane or total expression (Figure 4G,H).

These results indicated that TCA evokes direct extracellular inhibition of the TRPC4 current without changing TRPC4 expression. Therefore, TCA compounds absorbed into the gut have negative potential that is sufficient to broadly block TRPC4 functions in intestinal smooth muscle.

### 3.4 TCA remarkably suppresses the m*I*_cat_ formed by TRPC4 in isolated murine colonic myocytes

**FIGURE 5:**
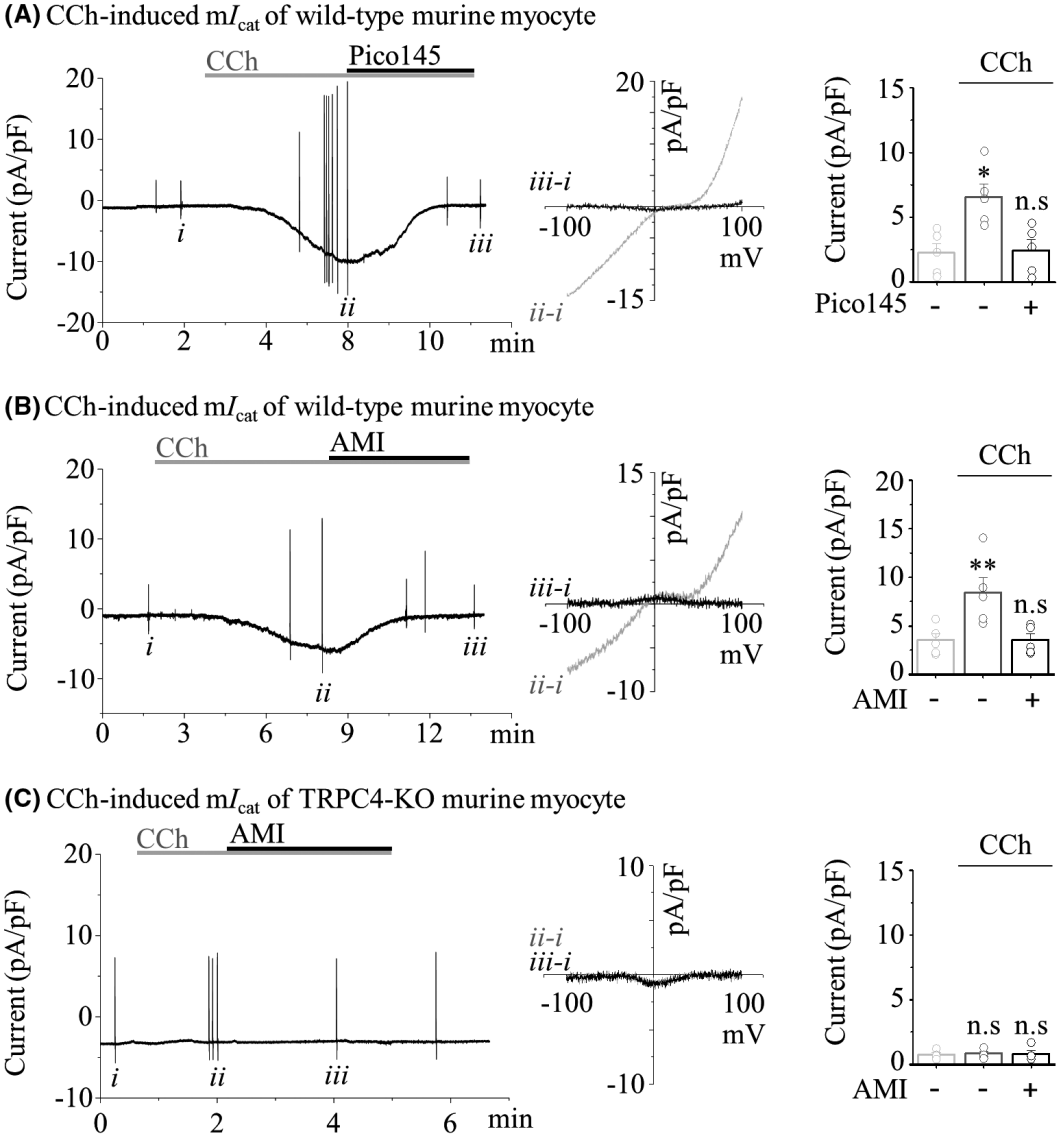
Inhibition of the CCh‐induced TRPC4 current by AMI in murine myocytes. In all panels, representative current trace (*left*) and IV curves (*middle*) showing the whole‐cell current in murine sigmoid colonic myocytes evoked by 100 μM CCh. Summarized data (*right*) showing the current density (pA/pF) at −60 mV. (A) 100 nM Pico145 (*n* = 5). (B and C) 10 μM AMI (*n* = 5 of B and *n* = 5 of C)

It is well defined that m*I*_cat_, observed in murine myocytes, is prominently elicited by a TRPC4‐mediated cationic current.^32^, ^51^ To further clarify whether TCA blocks the m*I*_cat_ of the colonic myocyte response to CCh, we prepared myocytes from murine sigmoid colon tissue by enzymatic isolation following our previous procedure.^52^ Under the optimized conditions of TRPC4 recording similar to that of TRPC4‐overexpressing HEK cells, the m*I*_cat_ from a single myocyte was recorded. We ensured that the newly established data recorded in colonic myocytes met the following standards (Figure 5A): (1) the current‐voltage relationship (*I*–*V* curve) of the CCh‐evoked inward current exhibited a typical doubly rectifying shape of TRPC4. (2) The selective and potent antagonist of TRPC4, Pico145, completely blocked the CCh‐evoked current. (3) In colonic myocytes obtained from TRPC4‐deficient mice, m*I*_cat_ was not observed. To determine whether TCA suppresses the m*I*_cat_ of colonic myocytes, we perfused TCA before or after the CCh‐evoked current. As shown in Figure 5B, AMI substantially inhibited the m*I*_cat_, which responded to CCh, to the basal current level. The m*I*_cat_ of TRPC4‐deficient colonic myocytes was not evoked by CCh at all, and interestingly, the basal current was not further suppressed by AMI (Figure 5C).

**FIGURE 6:**
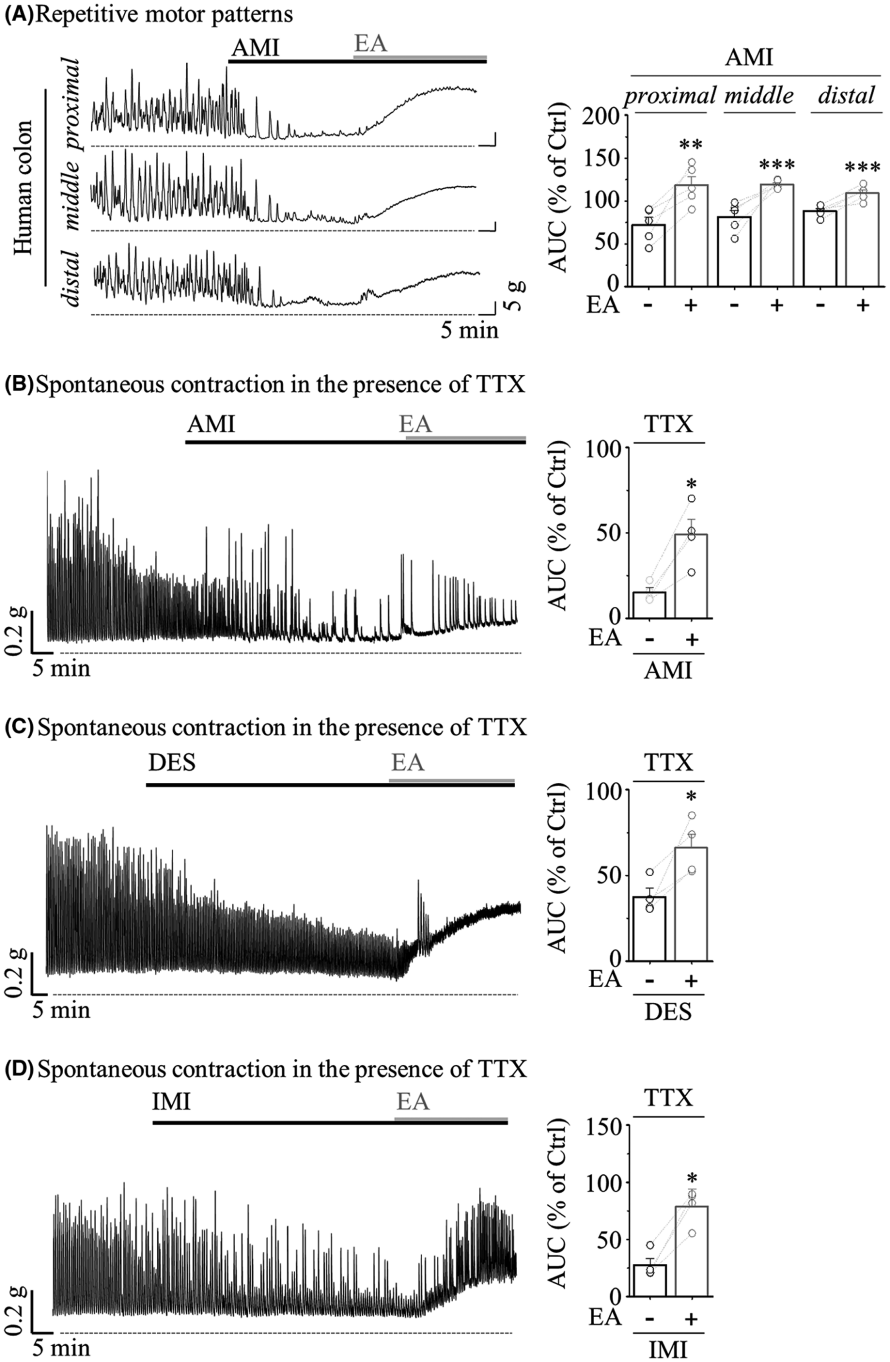
Restoration of TCA‐induced colonic motility inhibition against TRPC4 activation. Representative trace (*left*) and summarized bar graph (*right*) showing AUC induced by 1 μM EA (grey) after TCA treatment (black). (A) RMPs (*n* = 5). (B–D) Spontaneous contraction pretreated with 1 μM TTX for 10 min. (B) 10 μM AMI (*n* = 5). (C) 10 μM DES (*n* = 4). (D) 10 μM IMI (*n* = 5)

The following experiment was designed to evaluate whether TCA‐induced myocyte inactivation could be improved by modulating TRPC4 activity as a therapeutic approach for constipation. As shown in Figure 6A, potent inhibition of RMPs with reduced amplitude and frequency was rescued by TRPC4 activation with 10 nM EA. The higher concentration of 100 nM EA led to tonic contractions with a cumulative response in partial frequency recovery of the proximal (21.20 ± 5.38%), middle (25.81 ± 10.22%) and distal (32.62 ± 8.14%). Even under TTX pretreatment, the amplitude of spontaneous contractions, which was reduced to 15.28 ± 2.58% in AMI, was restored to 49.03 ± 8.84% by EA (Figure 6B). In addition, EA significantly improved the spontaneous contractions suppressed by DES and IMI (37.57 ± 4.95 to 66.19 ± 7.98% of DES, 27.68 ± 5.82 to 78.71 ± 15.44% of IMI, Figure 6C,D).

These results of Figure 5 indicate that the m*I*_cat_ suppressed by TCA is ultimately responsible for the inhibition of TRPC4 channels expressed in colonic myocytes. The results of Figure 6 mimicking the therapeutic evaluation of TCA‐induced constipation and IBS‐D indicate that colonic motility atrophied by TCA was improved by the restoration of TRPC4 activity.

## 4 DISCUSSION

The GI tract is made up of four layers: the innermost layer is the mucosa, underneath this is the submucosa, followed by the muscularis propria (muscular layer) and finally, the outermost layer—the adventitia (or serosa**)**. The muscular layer is made up of two layers of smooth muscle, the inner, circular layer, and the outer, longitudinal layer. The rhythmicity of segmentation requires a myogenic pacemaker that is evoked by synchronized reconstitution of the enteric motor neurons and ICC‐IM. In contrast to myogenic slow waves, whose frequency of depolarization is determined by ICC, mass peristalsis comprises an extensive region of sustained and coordinated contraction of proximal and distal smooth muscle segments that propagates rapidly by neurogenic contractions. RMPs recorded in colonic segment are propulsive contraction with high amplitude and low frequency. These are neurogenic contractions which are mediated by enteric neuron. Although our observations on TCA‐induced disturbed motility were limited to only the human sigmoid colon (Figures 1, 2, 3), this TCA action is ultimately responsible for the inhibition of TRPC4 channels expressed in colonic myocytes (Figures 4 and 5).

On the contrary, ICC‐mediated spontaneous contractions recorded in colonic strip are rhythmic contractions with low amplitude and high frequency. In a previous report, Nakayama et al. reported that SKF96365, a TRPC4 blocker, inhibited spontaneous contraction caused by ICC, and revealed that Ca^2+^ introduced by TRPC4 contributes to the action of RyR3, which is important for inducing pacemaker current.^53^, ^54^ Therefore, a discussion on the function of TRPC4 expressed in ICC cannot be excluded. However, each contraction contributes to optimal mixing and propulsion of the contents, sometimes separately and sometimes together. In these experiments, TCAs inhibited both RMPs and EFS‐induced contractions (Figures 1, 2A and Figure S2A). These results implicate that TCA acts on enteric nerves or directly on SMCs to inhibit colonic contractions. In addition, TCAs inhibited spontaneous contractions and it is not affected by TTX (Figure 2B–D and Figure S2C–F). Although the action of TCAs on enteric neurons and ICC cannot be ruled out, this result raises the possibility that TCAs act directly on smooth muscle to decrease colonic motility and consequently induce constipation.

Constipation is one of the most commonly reported adverse symptoms with many medications (anticholinergics, antihypertensives, antidepressants, iron supplements, narcotic analgesics and calcium channel blockers). Constipation is characterized by digested food wastes that absorb too much water to create a dry solid matter called stool or prolonged transit time of stool that moves slowly through the digestive tract due to poor GI motility.^55^, ^56^ According to the data of our patch clamp (Figures 4 and 5) and motility (Figures 1, 2, 3 and 6), TCAs might have a primary effect on myocyte activity because they are rapidly absorbed into the intestinal smooth muscle layer rather than systemic circulation due to the nature of oral administration. Thus, TCA compounds absorbed into the gut are sufficient to broadly block TRPC4 activity in intestinal smooth muscles prior to all nervous systems.

In various regulatory aspects of GI tract, it has been demonstrated through an amount of reports that the TRP channels have important functions.^57^, ^58^ Several TRP channels are involved in ENS signalling and are known to contribute to visceral sensitivity and hypersensitivity (TRPA1, V1 and V4).^59^, ^60^, ^61^ In addition, TRPM7 expressed in ICC is expected to contribute to intestinal pacemaking.^62^ In particular, TRPV1 antagonists are currently used as therapeutic agents for heartburn and visceral hypersensitivity, but various TRP channels are likely to be proposed as targets for intestinal diseases.^63^ In complete contrast to constipation, diarrhoea is the primary symptom in patients with IBS‐D, characterized by sudden urges to have bowel movements along with loose stools, frequent stools, abdominal pain and discomfort gas. Considering this, a therapeutic approach targeting TRPC4 can be more effective in ameliorating diarrhoea, such as IBS‐D symptoms. TRPC6 can also be a target in terms of improving colonic motility through inhibition of smooth muscle contraction, but its contribution in depolarization of myocyte is relatively lacking compared with TRPC4,^32^ and our results also showed an almost complete decrease in current in TRPC4 KO mice and blocker (Figure 5). Therefore, the TRPC4 channel should be considered a reasonable candidate as a molecular target of TCA‐induced constipation and IBS treatment with TCA. Although we believe that TRPC4 channel of colonic myocytes has functional potential as an alternative molecular target to treat IBS with TCA, it is worthy of further study using a humanized mouse model of IBS.

Given the psychiatric range used for antidepressant (100–200 mg/day) treatment,^34^ the dosage of TCAs used for IBS (25–125 mg/day)^21^ is considered to be low. Moreover, the concentrations we applied in the suppression of spontaneous RMP and myocyte activities by gut motility are likely to be higher than the estimated serum concentration (100–300 ng/ml) of TCA.^34^ Nevertheless, the pharmacological properties of TCAs can produce unintended biological activities via potential off‐target effects. In addition to TRPC4, previous studies have reported that various ion channels are inhibited by TCA, and in particular, ATP‐dependent K^+^ channels^52^ and L‐type calcium channels^64^ are importantly involved in ICC activation and myocyte contraction in the GI tract, so it is difficult to exclude the effect of TCA. As shown in the EFS‐induced contractile activity of supplementary Figure 3C,D, even after preinhibition of TRPC4 with Pico145, the amplitude that is partially suppressed by TCA remained. On the contrary, pretreatment with AMI was completely blocked even at higher frequencies. Likewise, we cannot rule out the possibility that it accounts for another target of AMI together with TRPC4 despite the apparent absence of TRPC4 activity by Pico145.

To date, the treatment of diarrhoea with antidepressants has relied on clinical statistics, and although the physiological mechanisms are not clearly understood, these findings conclude that TRPC4 is a critical regulator of the suppression of intestinal motility by TCA. Taken together, our new target, TRPC4, will provide clinical insights into medical interventions aimed at IBS, as well as expanding the understanding of various adverse effects of TCA.

## AUTHOR CONTRIBUTION

**Byeongseok Jeong:** Formal analysis (equal); Investigation (equal); Writing – original draft (equal). **Tae Sik Sung:** Conceptualization (equal); Data curation (equal); Formal analysis (equal); Investigation (equal). **Dongju Jeon:** Data curation (supporting); Validation (supporting). **Kyu Joo Park:** Methodology (supporting); Resources (supporting). **Jae Yeoul Jun:** Conceptualization (supporting); Funding acquisition (supporting); Methodology (supporting). **Insuk So:** Funding acquisition (supporting); Methodology (supporting); Supervision (supporting); Writing – review & editing (supporting). **Chansik Hong:** Conceptualization (lead); Funding acquisition (lead); Project administration (lead); Writing – original draft (equal).

## CONFLICT OF INTEREST

The authors declare that they have no competing interests.

## Supporting information

Fig S1

Fig S2

Fig S3

Fig S4

Supplementary Material

## Data Availability

The datasets used and/or analysed during the current study are available from the corresponding author upon reasonable request.
