# Supplementary figures and images for: Inhibition of TRPC4 channel activity in colonic myocytes by tricyclic antidepressants disrupts colonic motility causing constipation
Source: J Cell Mol Med. 2022 May 12;26(19):4911–23. doi: 10.1111/jcmm.17348 (PMC9549500; doi:10.1111/jcmm.17348)

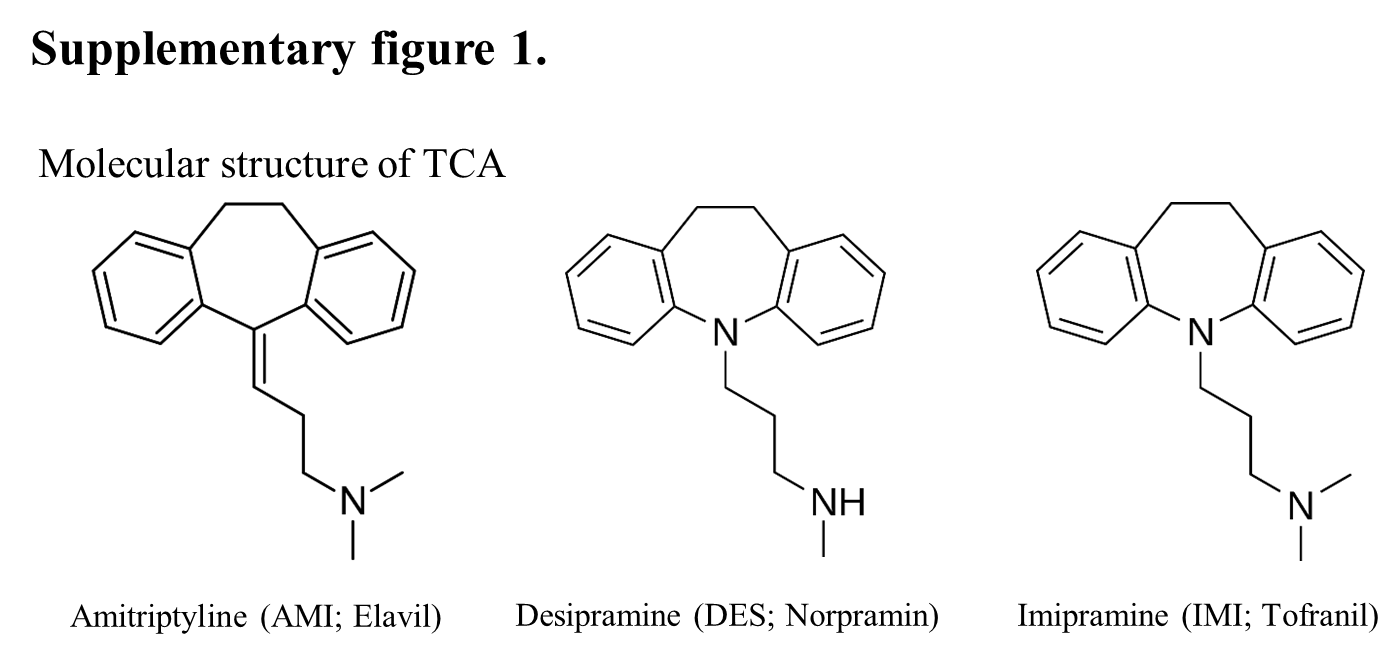

Supplement: Supplementary file 1 — Fig S1 [file JCMM-26-4911-s005.tif]

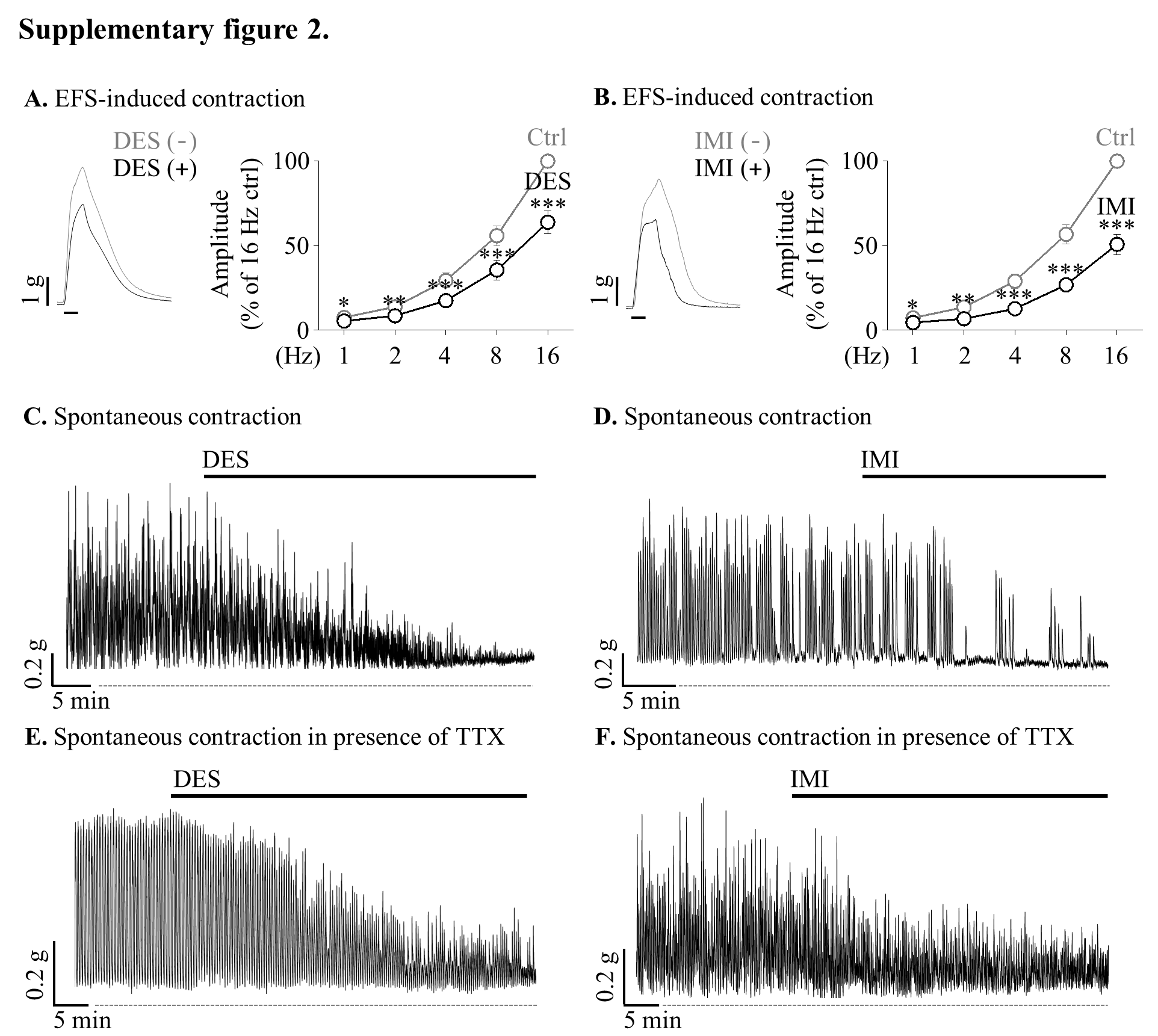

Supplement: Supplementary file 2 — Fig S2 [file JCMM-26-4911-s004.tif]

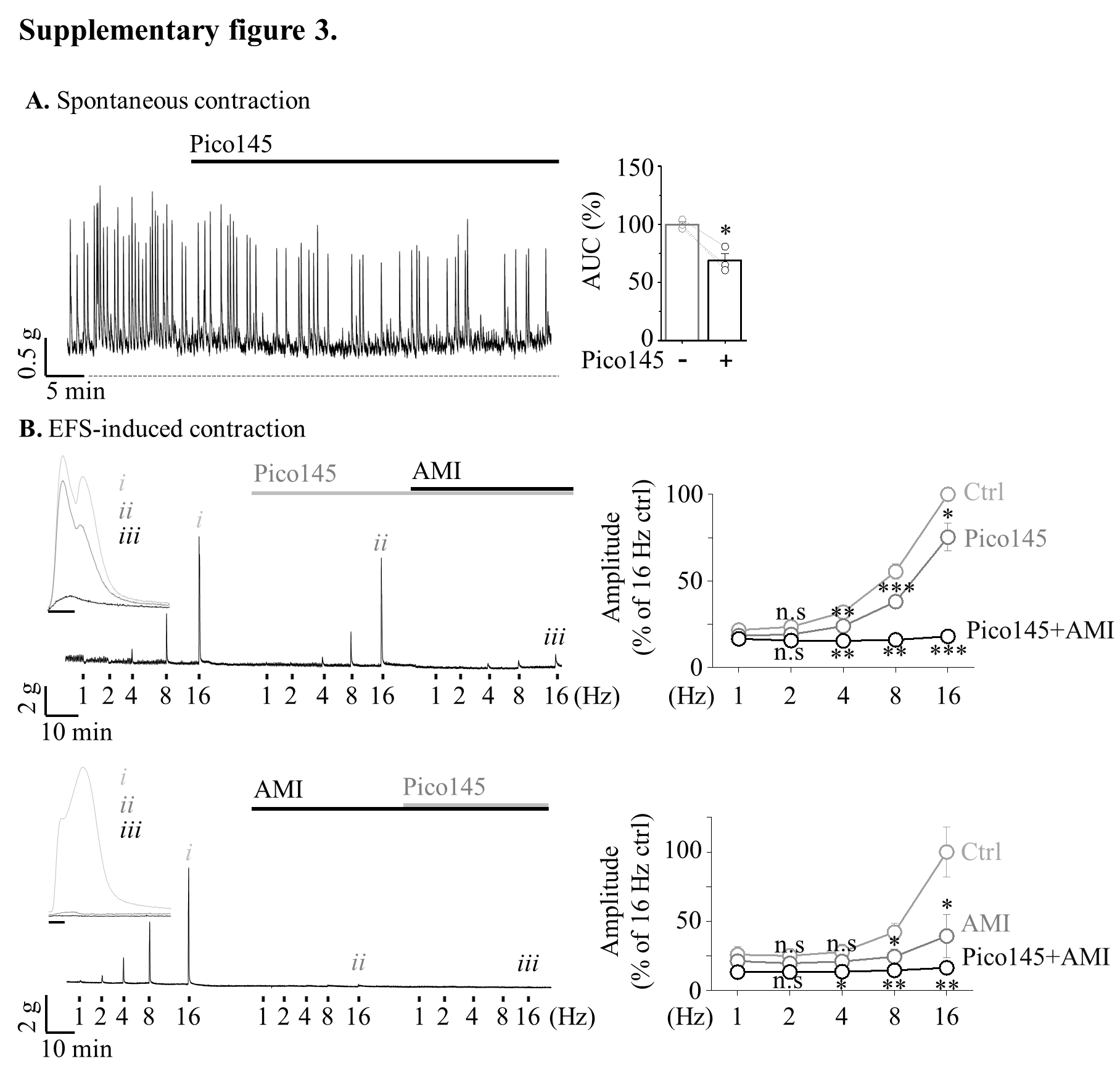

Supplement: Supplementary file 3 — Fig S3 [file JCMM-26-4911-s003.tif]

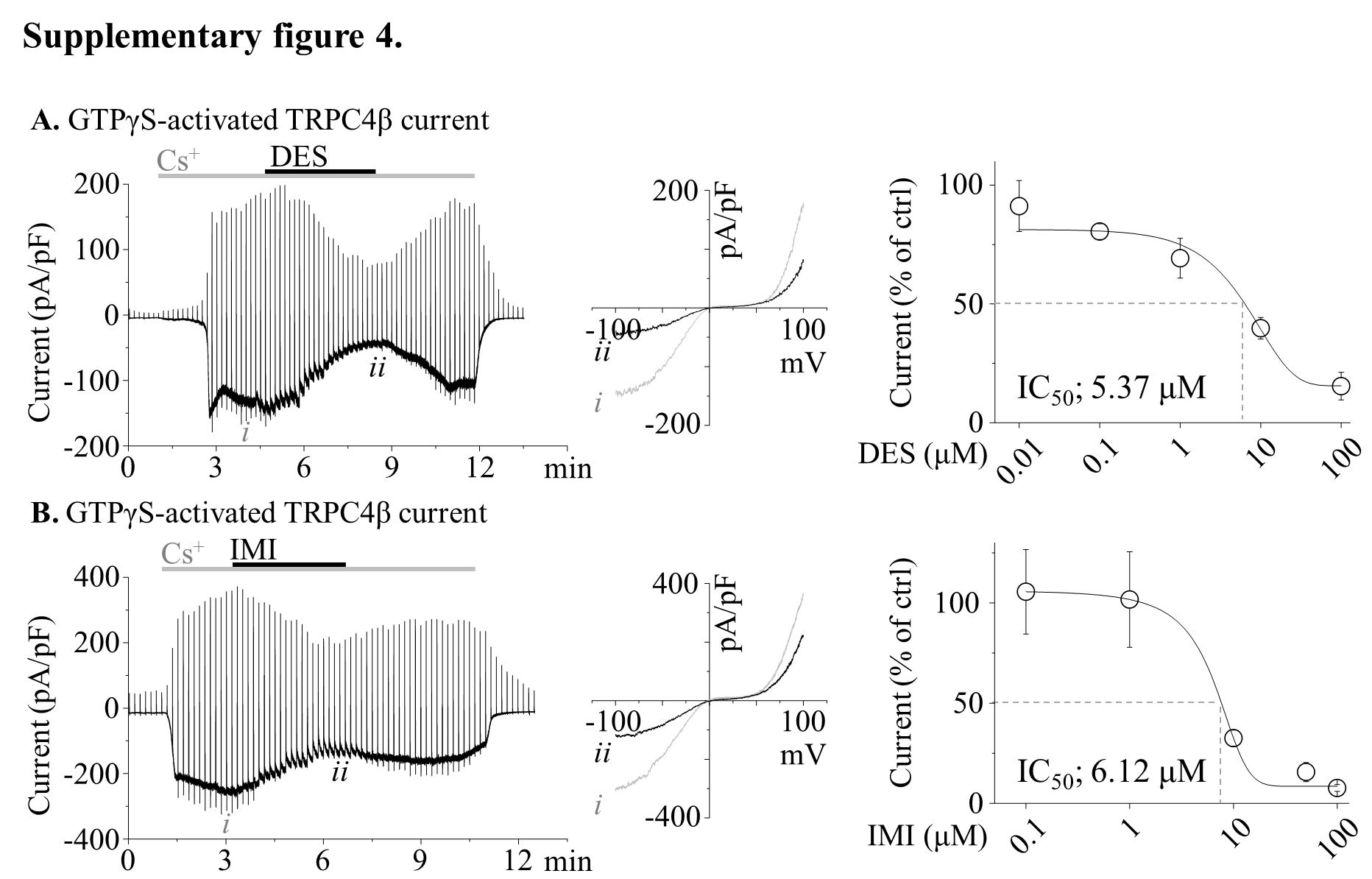

Supplement: Supplementary file 4 — Fig S4 [file JCMM-26-4911-s002.tif]
