# Supplementary material for: Inhibition of TRPC4 channel activity in colonic myocytes by tricyclic antidepressants disrupts colonic motility causing constipation
Source: J Cell Mol Med. 2022 May 12;26(19):4911–23. doi: 10.1111/jcmm.17348 (PMC9549500; doi:10.1111/jcmm.17348)
Supplement: Supplementary file 5 — Supplementary Material [file JCMM-26-4911-s001.docx]

**Title**

Inhibition of TRPC4 channel activity in colonic myocytes by tricyclic antidepressants disrupts colonic motility causing constipation

**Author Names**

Byeongseok Jeong^1,#^, Tae Sik Sung^2,3,#^, Dongju Jeon^1^, Kyu Joo Park^2^, Jae Yeoul Jun^1^, Insuk So^4^, Chansik Hong^1,*^

**Supplementary Figure Legends**

**Supplementary figure 1.**

Chemical structures of TCAs

**Supplementary figure 2.**

**A** and **B.** Inset traces (*left*) showing the contractile responses before (gray) and after (black) application of TCA. Summarized amplitude data (*right*) on the inhibition rate at 16 Hz. **C-F**. Representative traces of spontaneous contraction suppressed by TCA. **A**, **C** and **E.** 10 μM DES. **A.** n=7 **B**, **D** and **F.** 10 μM IMI. **B.** n=6

**Supplementary figure 3.**

**A.** Representative trace (*left*) and summarized data of spontaneous contraction induced by 100 nM Pico145 (n=3). **B.** Representative traces (*left*) and summarized amplitude data of EFS-induced contraction suppressed by 10 μM AMI and 100 nM Pico145 (n=5).

**Supplementary figure 4.**

In all panels, representative current traces (*left*) and IV curves (*middle*) of 200 μM GTPγS-activated TRPC4β current reduced by 10 μM TCAs. The dose-dependent curves (*right*) of the inhibition rate depending on the TCA concentration. **A**. DES. **B**. IMI.
